# The relationship between dental anxiety and other kinds of anxiety: a naturalistic, cross-sectional and comparative study

**DOI:** 10.1186/s40359-021-00684-6

**Published:** 2021-11-24

**Authors:** Juan Valdes-Stauber, Kevin Hummel

**Affiliations:** Department of Psychiatry and Psychotherapy I, University of Ulm, Ulm, Germany

**Keywords:** Dental anxiety, Odontophobia, Self-efficacy, Loneliness, Neuroticism

## Abstract

**Background:**

Dental anxiety is of public health importance because it leads to postponed dental treatment, which comes with health complications. The present study investigated whether there is a correlation between the degree of dental anxiety and other kinds of anxiety and whether there are prognostic factors for the different kinds of anxiety.

**Method:**

In the sample (N = 156) from a dental practice in a large German city, 62% of patients received a check-examination and 38% received dental surgery. The target variables were recorded with validated questionnaires: dental anxiety (IDAF-4c+), subclinical anxiety (SubA), anxiety of negative evaluation (SANB-5), current general anxiety (STAI state), loneliness (LS-S) and self-efficacy (GSW-6). The applied statistics were: t-tests for 31 variables, correlation matrix and multivariate and bivariate regression analyses.

**Results:**

The dental surgery patients displayed more dental anxiety and more dental interventions than the check-examination group. The main result was a positive correlation of all kinds of anxiety with each other, a positive correlation of loneliness and neuroticism with all forms of anxiety and a negative correlation between all forms of anxiety and self-efficacy. Especially dental anxiety is positively associated with other kinds of anxiety. In multivariate regression models only neuroticism is associated with dental anxiety, but feelings of loneliness are positively associated with with the other kinds of anxiety assessed in this study. The higher the self-efficacy, the lower the level of general anxiety.

**Conclusions:**

In dentistry, anxiety from negative experiences with buccal interventions should be distinguished from anxiety caused by personality traits. Self-efficacy tends to protect against anxiety, while loneliness and neuroticism are direct or indirect risk factors for anxiety in this urban dentistry sample. Dental anxiety seems to be independent from biographical strains but not from neuroticism. In practice, more attention must be paid to anxiety control, self-management and efforts to improve the confidence of patients with emotional lability, less self-confidence and propensity to shame.

**Supplementary Information:**

The online version contains supplementary material available at 10.1186/s40359-021-00684-6.

## Background

Dental health is a keystone of systemic health and quality of life [1, 2], as demonstrated by dental paleoanthropology [3, 4]. Postponing dental treatment and avoiding dentists because of dental anxiety and fear [5] or socio-economic barriers are relevant health factors [6], even as a mediator between one’s psychopathological burden and childhood caries [7]. According to World Health Organization (WHO) estimates, dental anxiety and fear affects 15–20% of the worldwide population, a figure that is of interest for public health because dental anxiety increases the risk of neglecting dental health [5]. The overarching concept is “odontophobia”, which encompasses “dental anxiety”, “dental fear” and “dental phobia” as synonyms but sometimes also as degrees of odontophobia. This concept includes avoidance of indispensable dental treatment, as recognized by Corah et al. [8, 9]. There are validated scales to assess different aspects of dental anxiety [8, 10, 11].

Dental anxiety has traditionally been seen as a separate, independent anxiety caused by bad dental experiences [12]. This view is in line with classical conditioning mediated by anxiety-provoking stimuli like an anaesthesia syringe, noisy drill instruments, or other dental equipment. Therefore, a range of techniques has been introduced in dental medicine in order to gain the patient’s trust and mitigate anxiety [13]. According to a Finnish survey, among anxiety factors, anticipatory anxiety is particularly related to clinical anxiety and depression, especially in males. In females, anticipatory anxiety is also related to treatment dental anxiety [14]. There is increasing evidence that severe anxiety in conjunction with phobia is related to other kinds of anxiety as well as mental disorders, e.g. depression, anxiety disorders and especially post-traumatic stress disorders [13, 15]. Therefore, traumatic experiences are a serious risk factor for dental anxiety, fear, and phobia [16]. Overall, people with severe mental disorders like schizophrenia show high levels of unmet dental needs [17, 18]. However, increasing attention is paid not only to dental patients with anxiety, especially heavily burdened patients, but also to the risk of burnout by dentists themselves due to emotional dysregulation and the social cognitive function of empathy with patients [19, 20].

The present investigation examined the hypothesis that dental anxiety is associated with other kinds of anxiety due to a possible common factor beyond a classical negative conditioning in dental anxiety. The survey was conducted in Germany; therefore, some German epidemiological data must be highlighted. In Germany, every citizen visited the dentist on average 1.5 times in 2018, including 1.4 surgical-conservation consultations; the number of visits has risen slightly but steadily every year over the past 25 years [21]. In Germany, 48% of people over the age of 16 forego a visit to the dentist, even though it would be necessary; a third of these individuals do not go to the dentist for financial reasons [22]. The caries index (decayed, missing and filled teeth per 28 teeth) for the 35–44-year-old group is 14.5 [23].

In line with the literature, the present study further examines whether there are associations between personality traits (especially neuroticism) and adverse biographical conditions or personal resources like self-efficacy or respective risks such as loneliness with dental anxiety.

The principal purpose of the study was to assess possible associations between dental anxiety and other kinds of anxiety as well as possible prognostic factors for different kinds of anxiety in an unselected German urban sample attended in dental offices. The results could focus greater awareness among dental and public health practitioners of anxiety in dentistry, especially considering risk features.

## Methods

### Objective and study design

The main objective was to assess possible associations between dental anxiety and other kinds of anxiety as well as personality traits, including those considered as a resource (self-efficacy) and a risk (loneliness). Since the study goes beyond the collection of routine data, approval was obtained from the ethics committee of the University of Ulm (registration number 285/17). Written informed consent in German was obtained from all participants. The authors started from the hypothesis that dental anxiety arises as a result of classical conditioning by a negative dental experience, as well as life history and personality variables that influence the extent of odontophobia. This main objective includes the following single questions:

- What is the socio-demographic, personality-related, medical and biographical profile of a dentistry unselected sample in a German urban area?
- Is there a profile difference between individuals who come to the dentistry office for a check-examination and those who are electively examined because of an acute dental problem?
- Are there correlations between personality traits, dental fear, other kinds of anxiety, personal resources (self-efficacy) and risks (loneliness)?
- Are there associations between dental anxiety and other kinds of anxiety?
- Are there prognostic factors among selected variables for dental fear as well as for other kinds of anxiety in a sample of dentistry patients?

This prospective, naturalistic cross-sectional study was conducted in two metropolitan dental offices to investigate the connections between dental anxiety and other forms of anxiety. The sample (N = 156) was not selected, i.e. there were no further inclusion or exclusion criteria other than informed consent. Fifteen patients (average age 49 years; 53% dental check-up, 47% indicative dental intervention; 60% women) did not consent to the study. The participants were divided into two naturalistic groups: (a) oral health control (62%) and (b) indicative dental intervention (38%).

To determine the psychosomatic correlations with regard to the objective, the following groups of variables were examined (see Tables 1 and 2):

- socio-demographic variables: age, sex, living alone or not, education level, employment status;
- dental variables: age at onset of dental problems, dental appointments last 12 months, different dentists during the lifetime;
- variables for somatic health burden: number of different drugs currently, medical appointments last 12 months;
- biographical variables (as an indication of the mental health burden): any psychiatric support during the lifetime, appraisal of one’s life and upbringing (scale 0–10), traumatic experiences in childhood and as an adult (dichotomous);
- resources as an indication of structural resilience (self-efficacy) or of vulnerability (loneliness);
- personality dimensions as robust features of personality, measured with the Big Five Inventory (BFI-10): extraversion, agreeableness, conscientiousness, neuroticism, openness;
- anxiety kinds: anxiety as personality trait; current anxiety level; fear of negative evaluation by others, and dental anxiety.

**Table 1:** Multidimensional description of the sample

	Whole sample						Comparison between check-examination group (0) and dental treatment group (1) (t- test or chi-square test)		
	N	M or %	(SD)	Vc	Median	Range	t or chi^2^	p	Cohen’s d or Cramer’s V
*Socio-demographic variables*									
Age	156	51.2	(17.7)	35%	55	17–87	0.77	n.s	0.14
Sex (% women)	156	57%					0.001	n.s	0.003
Living alone	155	32.3%					3.22	n.s	0.16
Education (% high school)	154	68%					1.38	n.s	0.10
Employment (% employed/jobless/pension)	154	67/1.3/21%					3.95	n.s	0.17
*Odontological variables*									
Age at onset of dental problems	127	13.0	(7.3)	56%	11	3–50	0.99	n.s	0.04
Dental appointments in the last 12 months	155	2.8	(2.6)	93%	2	0–25	2.65	0.009	0.48
Different dentists during the lifetime	152	6.0	(5.3)	88%	5	1–40	1.46	n.s	0.26
*Medical variables*									
Number different drugs currently	156	1.1	(1.7)	155%	0	0–8	0.66	n.s	0.12
Medical appointments last 12 months	156	5.4	(5.4)	100%	4	0–40	0.44	n.s	0.08
Number physical illnesses	156	0.77	(0.97)	126%	0	0–4	0.74	n.s	0.13
*Biographical data*									
Any psychiatric support during the lifetime	155	39.3%					2.14	n.s	0.13
Appraisal of one’s life	155	7.9	(1.4)	18%	8	2–10	0.04	n.s	0.01
Appraisal of one’s upbringing	154	7.5	(2.1)	28%	8	1–10	0.74	n.s	0.13
Traumatic experience in childhood	155	45.8%					0.14	n.s	0.03
Traumatic experience as an adult	156	35.9%					0.01	n.s	0.01

N = sample size; M = mean; SD = standard deviation; Range = lowest and highest value; Vc = variation coefficient: (SD/M) × 100; t = t- value on a t-distribution; chi^2^ = value at chi-square distribution of values; p = level of significance of differences; Cohen’s d = effect size of differences for metric variables; Cramer’s V = effect size of differences for categorical variables

**Table 2:** Psychosomatic profile of assessed sample

	Whole sample						Comparison between check-examination group (0) and dental treatment group (1) (t-tests)		
	N	M	(SD)	Vc	Median	Range	t	p	Cohen’s d
*Personality (BFI-10)*									
Extraversion	152	3.41	(0.97)	28%	3.50	1–5	1.06	n.s	0.19
Agreeableness	152	3.37	(0.73)	22%	3.50	1.5–5	0.76	n.s	0.14
Conscientiousness	152	4.07	(0.68)	17%	4.00	2–5	1.29	n.s	0.24
Neuroticism	150	2.96	(1.08)	36%	3.00	1–5	0.14	n.s	0.03
Openness	151	3.70	(0.90)	24%	4.00	1–5	0.75	n.s	0.14
*Resources*									
Self-efficacy (GSW-6)	155	18.4	(2.7)	15%	18	9–24	1.52	n.s	0.20
Loneliness (LS-S)	155	5.9	(2.0)	34%	6	3–14	1.57	n.s	0.28
*Anxiety profile*									
Anxiety traits in personality (Sub A)	155	2.5	(1.0)	40%	2.4	1–5.6	1.09	n.s	0.20
Fear of negative evaluation by others (SANB-5)	156	9.1	(3.0)	33%	9	5–20	0.94	n.s	0.17
Current anxiety (STAI state)	151	35.3	(10.9)	31%	34	5–69	0.48	n.s	0.09
Dental anxiety and fear (IDAF-4c +)	154	1.7	(0.8)	47%	1.4	1–5	2.61	0.010	0.47
Emotionality	154	1.9	(1.1)	58%	1.5	1–5	2.81	0.006	0.51
Behaviour	154	1.8	(1.0)	55%	1.5	1–5	1.73	n.s	0.31
Physiology	154	1.7	(1.1)	65%	1.0	1–5	2.63	0.020	0.43
Cognition	154	1.4	(0.7)	50%	1.0	1–5	1.84	n.s	0.33

N = sample size with index variable information; M = mean; SD = standard deviation; Range = lowest and highest value; Vc = variation coefficient: (SD/M) × 100; t = t-value on a t-distribution; p = level of significance of differences; Cohen’s d = effect size of differences for metric variables; BFI-10 = Big-Five Inventory, 10 items; GSW-6 = Generalisierte Selbstwirksamkeit (generalized self-efficacy); LS-S = Loneliness Scale-short version; Sub A = Skala Subklinische Angst (subclinical anxiety scale); SANB-5 = Skala Angst vor negative Bewertung (short version of Fear of Negative Evaluation Scale); STAI = State-Trait-Anxiety-Inventory, state subscale; IDAC-4c+ = Index of Dental Anxiety and Fear-four dimensions

Dental anxiety was examined on four levels (emotional, behavioural, physiological and cognitive) and measured with the German version of the IDAF-4c+. Subclinical anxiety as an indicator of personality disposition was measured with the Subclinical Anxiety (SubA) scale. Current anxiety was measured with the state part of the State Trait Anxiety Inventory (STAI). Finally, a personality and biography-relevant form of social anxiety, namely the fear of evaluation/rejection, was measured with the scale Fear facing Negative Evaluation (SANB-5) (see Table 2).

Validated short psychometric tools were used:

- Big-Five-Inventory, 10 items (BFI-10): good objectivity, re-test reliability after 6 weeks (between 0.49 and 0.62), good convergent validity as well as factor and construct validity [24];
- Loneliness Scale-Short version (LS-S): psychometric assessments: Cronbach’s α = 0.97, positive correlation with neuroticism (r = 0.27), negative with extraversion (r = − 0.16), well-being (r = − 0.25) and life satisfaction (r = − 0.43) [25–27];
- Generalized Self-Efficacy (GSW-6); psychometric assessments: Cronbach’s α for a sample of patients with heart failure 0.86; test–retest reliability after 12 months 0.50, after 28 months 0.60; negative association with depressivity (r = − 0.45), anxiety (r = − 0.35) and vital exhaustion (r = − 0.35), and positive with mental health (r = 0.36) [28];
- Subclinical anxiety (SubA): Internal consistence (Cronbach’s α) between 0.64 and 0.84; good convergent validity with impairment of well-being (r = 0.33 to 0.55) and depressivity (r = 0.53 to 0.56); negative association with self-esteem and feeling of control (r = − 0.22 to − 0.42) [29, 30];
- Negative Assessment Scale (SANB-5): Cronbach’s α = 0.84 to 0.94; positive correlation with test anxiety (r = 0.37) and negative with life satisfaction (r = − 0.44) [31–33];
- State-Trait-Anxiety-Inventory, module state STAI-S: very good internal consistency (r = 0.90), re-test validity for state module after 63 days between r = 0.22 and r = 0.53, good convergent and divergent validity with several scales [34–37].
- The IDAF-4c + comprises eight items that correspond to four dental anxiety dimensions: emotional, cognitive, physiological and behaviour [10, 38]. The average score for the German sample assessed by Tönnies et al. was 2.47 (standard deviation [SD] = 1.31) for women and 2.13 (SD = 1.10) for men [10]. Internal consistency for the basic module was α = 0.97, test–retest after 4 months r = 0.82, and good convergent validity with DAS (r = 0.87) and DFS (r = 0.92) [10].

Internal consistency assessed for this sample: GSW-6 (α = 0.83); SANB-5 (α = 0.87); SubA (α = 0.76); LS-S (α = 0.80); STAI, module state (α = 0.93), and IDAF-4c, basic module (α = 0.93).

### Statistics

The first question was assessed by means of descriptive statistics applied to the target variables and the profile variables (see Tables 1 and 2).

The two treatment groups were compared for each metric variable using the parametric t-test for unpaired samples and for the nominal variables using the chi-square test. By increasing the sample size, statistical tests are robust to normality; therefore parametric tests to assess differences were implemented. The effect sizes of the differences were determined using Cohen’s d (for metric variables) or Cramer’s V (for nominal variables; Tables 1 and 2). For Cohen’s d, 0.2 to 0.5 means a small effect, 0.5 to 0.8 a medium effect, and > 0.8 a strong effect (see Tables 1 and 2).

The correlation between personality dimensions, resources and kinds of anxiety was investigated using a correlation matrix with Pearson correlation coefficients and the significance level of the correlations. The effect strength for the Pearson correlations was defined as follows: 0.10–0.30 (small); 0.30–0.50 (medium); and > 0.50 (high) (see Table 3). The designations for Cramer’s V were similar to Pearson correlations.

**Table 3:** Correlation matrix between personality, resources and anxiety assessment instruments for the whole sample (N = 156)

	LS-S	GSW-6	SubA	SANB-5	STAI state	IDAF-4c
	r^p^	r^p^	r^p^	r^p^	r^p^	r^p^
Loneliness (LS-S)	–					
Self-efficacy (GSW-6)	− 0.21*	–				
Anxiety traits in personality (Sub A)	0.40***	− 0.54***	–			
Fear of negative evaluation by others (SANB-5)	0.51***	− 0.42***	0.62***	–		
Current anxiety (STAI state)	0.37***	− 0.40***	0.30***	0.47***	–	
Dental anxiety and fear (IDAF-4c+)	0.22**	− 0.29**	0.38***	0.38***	0.53***	–
BFI-Extraversion	− 0.19*	0.33***	− 0.30**	n.s	n.s	n.s
BFI-Agreeableness	n.s	n.s	n.s	n.s	n.s	n.s
BFI-Conscientiousness	− 0.17*	0.37***	− 0.41***	n.s	n.s	n.s
BFI-Neuroticism	0.28**	− 0.32**	0.30***	0.34***	0.29***	n.s
BFI-Openness	0.17*	0.20*	n.s	n.s	n.s	n.s

LS-S = Loneliness Scale-short version; GSW-6 = Generalisierte Selbstwirksamkeit (generalized self-efficacy); SubA = Skala Subklinische Angst (subclinical anxiety scale); SANB-5 = Skala Angst vor negative Bewertung (short version of Fear of Negative Evaluation Scale); STAI = State-Trait-Anxiety-Inventory, state subscale; IDAF-4c = Index of Dental Anxiety and Fear-four dimensions; BFI = Big-Five Inventory, 10 items; * < 0.05; ** < 0.01; *** < 0.001

The fourth question concerning simple associations between dental fear and other kinds of fear was investigated using bivariate regression models that are portrayed as scatter plots in order to appreciate individual differences and their dispersion (see Diagram 1).

The last question about possible prognostic factors for different kinds of fear in a dentistry sample was investigated by means of multivariate linear regression models with robust standard errors in order to calculate more accurate confidence intervals. The explained variance of the dependent variable through the model is given by a pseudo R^2^. The dependent variables were the four proposed kinds of anxiety and fear as the primary endpoints of the investigation. The independent variables (regressors) were chosen by following specific criteria: the two primary group comparisons (check-examination and dental treatment group); age and sex as basic socio-demographic variables; age of onset for dental problems as a surrogate variable for the burden of dental history; appraisal of one’s life and upbringing as well as trauma in childhood as indicators of biographical adversity at least from a first-person perspective; neuroticism as the most clinically relevant personality dimension; and self-efficacy as a positive and loneliness as a negative resource (see Table 4).

**Table 4:** Multivariate linear regression models with kinds of anxiety as the dependent variable using robust standard errors

	IDAF-4c+			SubA			STAI-state			SANB-5		
	Coef	t	*p*	Coef	t	*p*	Coef	t	*p*	Coef	t	*p*
Reason for consultation			n.s			n.s			n.s			n.s
Age			n.s	− 0.01	− 2.91	0.005			n.s			n.s
Sex			n.s			n.s			n.s			n.s
Age onset dental problems			n.s	0.04	2,26	0.026			n.s			n.s
Appraisal of one’s life			n.s			n.s			n.s			n.s
Appraisal of one’s upbringing			n.s	− 0.90	− 2,60	0.011			n.s			n.s
Trauma at childhood			n.s			n.s			n.s			n.s
Neuroticism	0.14	2.12	0.037			n.s			n.s			n.s
Self-efficacy (GSW-6)			n.s	− 0.13	− 4.30	< .001	− 1.01	− 2.56	0.012			n.s
Loneliness (LS-S)			n.s	0.14	3.13	0.002	1.79	3.04	0.003	0.62	3,06	0.003
*N*	98			99			96			99		
*F/p*	2.7/0.007			11.5/< 0.0001			4.1/< 0.0001			7.7/< 0.0001		
*R*^*2*^	0.17			0.51			0.37			0.46		

LS-S = Loneliness Scale-short version; GSW-6 = Generalisierte Selbstwirksamkeit (generalized self-efficacy); SubA = Skala Subklinische Angst (subclinical anxiety scale); SANB-5 = Skala Angst vor negative Bewertung (short version of the Fear of Negative Evaluation Scale); STAI = State-Trait-Anxiety-Inventory, state subscale; IDAF-4c+ = Index of Dental Anxiety and Fear-four dimensions; Coef = regression coefficient; t = t-value on a t-distribution; p = level of significance of the controlled association between the index independent and dependent variables; N = sample size encompassing all cases with full data in model; F = F-value for significance of whole model; R^2^ = proportion of variance of dependent variable explained by whole model

All statistical tests were conducted using the STATA.13 package.

## Results

The average age of the participants was 51 years; there were slightly more women (57%) than men. Over two thirds (68%) had completed secondary school, one third lived alone (32%) and only two respondents were unemployed (1.3%). On average, dental problems began at the age of 13. In the previous year, the subjects visited the dentist on average almost three times. The number of diagnoses, doctor’s appointments and medications were subject to a greater variance. Between one third and half of participants reported having suffered a traumatic experience. The global assessment of life and education was high (almost 8 on a scale of 0–10; Table 1), the personality had higher values for neuroticism and openness, while self-efficacy was moderately high and loneliness was low with minimal variation. Dental fear was rather low (1.7 out of a maximum of 5), and fear of rejection was moderately high on average (9.1 out of a maximum of 20) compared to the current fear (35 out of a maximum of 80) and fear traits in personality (2.5 out of a maximum of 21; Table 2).

Except for the dental and medical variables, the metric variables showed little to moderate variation. Apart from the personality dimensions, no metric variables were normally distributed. Therefore, the check-examination and dental treatment groups were compared with the nonparametric Mann–Whitney U test. There were very few statistical differences: the dental treatment group displayed more dental appointments in the past 12 months (3.6 vs. 2.3) and higher levels of dental anxiety (1.9 vs. 1.5), especially for the dimension “emotionality” (2.2 vs. 1.6; Tables 1 and 2).

The correlation study between personality dimensions, resources and anxiety forms showed a highly significant positive association between loneliness and all forms of studied anxiety, especially fear of evaluation/being rejected (r = 0.51). In contrast to this finding, the association between self-efficacy and all forms of anxiety was negative. All forms of anxiety correlated highly with each other (r > 0.30), especially subclinical anxiety and fear of rejection (r = 0.62), as well as fear of dental treatment and current level of anxiety (r = 0.53). In terms of personality dimensions, extraversion and conscientiousness correlated negatively with loneliness and subclinical fear and positively with self-efficacy; neuroticism correlated positively with all forms of fear (except fear of dental treatment) and with loneliness and negatively with self-efficacy (Table 3).

**Fig. 1:**
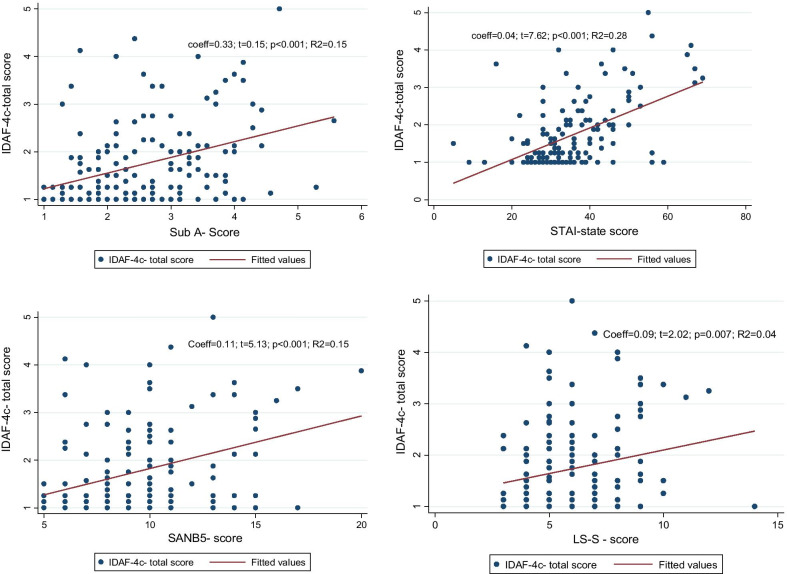
Associations between dental anxiety and other kinds of anxiety. In the top left between dental anxiety and subclinical anxiety. In the top right between dental anxiety and current anxiety; in the bottom left between dental anxiety and fear for negative evaluation; in the bottom right between dental anxiety and feeling of loneliness. Parameters: Coeff. = Robust estimator; t = value on a t-distribution; R2 = Coefficient of determination (square of correlation coefficient) as model fit parameter

Bivariate regression analyses displayed a statistically significant association between dental fear and other kinds of anxiety, especially for current anxiety (STAI state), but also between loneliness and dental fear. These results displayed graphically the results of the correlation matrix at an individual level, considering that the coefficient of determination of bivariate regression models is the square of the correlation coefficient (see Fig. 1).

By means of linear multivariate regression models, few prognostic factors for fear can be identified. Only higher scores of neuroticism were associated with stronger dental fear. For the other kinds of anxiety in the assessed dentistry sample, only self-efficacy was negatively associated with subclinical and current clinical anxiety; conversely, loneliness was overall associated with the investigated kinds of fear. Of importance are the results of the subclinical model since the coefficient of determination explained 51% of variance of the dependent variable. In this model, age was negatively associated with fear (t = − 2.91; p = 0.005), and the biographic appraisal of one’s upbringing was negatively associated with anxiety (t = − 2.60; p = 0.011) (see Table 4).

## Discussion

The main results of the study are the quite high rate of dentistry patients considering traumatic experiences in childhood and adulthood, but on the contrary reporting good appraisal of one’s life and upbringing that are not associated with general anxiety and dental anxiety with the exception of subclinical anxiety. There were no significant differences between individuals receiving dental check-examination and individuals attended because of acute dental problems except more dental anxiety in the last group. All kinds of anxiety were correlated with each other, and self-efficacy was negatively associated with anxiety, contrary to loneliness.

From a psychosomatic perspective, the self-efficacy rating in this study was similarly high for a population-based study (18.1 vs. 17.4) [28]; the feeling of loneliness was on average only “sometimes”. The dental anxiety was significantly lower than in the German validation of the IDAF-4C + (1.7 vs. 2.47 for women and 2.13 for men) [cf. 10]. Compared to population studies, fear as a personality trait (2.5 vs. 2.91) [cf. 30] and fear of negative evaluation (9.1 vs. 10.7) [cf. 33] were lower in the current study, while the state fear was below the cut-off value for clinically relevant fear (35 vs. 40) [cf. 37]. Overall, compared with the general population, the present sample was older, better educated, had a higher level of openness, was healthier than the national average and had lower dental anxiety. Notably, the self-efficacy and other kinds of anxiety in this sample were similar to the general population [cf. 39, 40]. Openness is congruent with quite a positive assessment of life, while neuroticism is possibly congruent with having experienced traumas, at least from a very subjective perspective. In this respect, the sample—which was recruited from an urban region—cannot be considered representative of the German population.

Regardless of the other variables, all the studied anxiety kinds, including dental anxiety, were highly significantly correlated with each other, as already noted in other studies [10, 41, 42]. This study found a positive correlation with feelings of loneliness and a negative correlation with self-efficacy for all kinds of assessed anxiety. These findings indicate that, on the one hand, all anxiety kinds may have common characteristics, and feelings of loneliness and lack of self-efficacy might be due to a common anthropological phenomenon as a general mediator like “self-esteem” or “overall confidence”. A causal direction cannot be assumed, but this factor could be investigated by means of mediation paths in further structural equation modelling. Similar interrelations have been found by Vigu and Stanciu [43].

In multivariate regression models, few variables showed an independent association with each assessed kind of anxiety; especially for dental anxiety, there was no association between it and the other kinds of anxiety. Loneliness and self-efficacy were the best predictors for anxiety, whereas neuroticism showed no association (with the exception of dental anxiety), contrary to results of bivariate correlations reported in other investigations [44]. These results could indicate that in fact dental fear is independent of biographical stress and rather dependent on emotional lability and conditioned by negative circumstances in dental treatment, as assumed in the literature. The other kinds of anxiety could be influenced by other factors than dental fear or could be part of deeper reasons for both interpersonal difficulties and less feeling of self-worth or self-esteem, leading to psychosocial arrangements which can be accompanied by loneliness.

Implications for the practice are the awareness of the importance of adverse psychosocial conditions and psychological features like self-esteem, shame and tendency to isolation as well as emotional lability in dental health, for example in avoiding dental treatment and in narrower compliance. Dentists have to focus on people with psychosocial disadvantages and with poorer psychological resources in order to improve adherence to treatment.

Limitations of the study are the narrow scope of dentistry patients that were not heavily impaired and were recruited from a well-positioned and little sickened middle-class population. There probably are mediators between anxiety and biographical as well as psychological features of importance, but such mediators have yet to be hypothesized. This is perhaps the reason why findings in the literature about the importance of adverse biography and psychosocial conditions were not associated with anxiety in this sample. Causal relationships cannot be established because the sample size is too small for calculations with latent variables in structural equation modelling.

## Conclusions

According to the results of this study, it should be of interest with regard to a more individualized dental medicine that dental anxiety is associated with other forms of anxiety, although there are hardly any prognostic factors for dental anxiety, at least in this urban, well-situated German sample. In contrast, age and self-efficacy appear to be protective factors, and neuroticism and feelings of loneliness appear to be risk factors for anxiety. For further health care-related studies, it would be interesting to investigate the extent to which dental anxiety is associated with other factors that are detrimental to health, for which further settings such as general practitioners or psychiatric homes would have to be investigated. The investigation of further risk and protective factors to more accurately assessing the psychological profiles are of epidemiological interest. But above all, a community-oriented dentistry has to investigate measures to increase accessibility, motivation for treatment readiness and the alleviation of anxiety in people suffering from dental anxiety in combination with other psychological burden.

## Supplementary Information

**Additional file 1**. Row individual data of the study according to assessed variables.

## Data Availability

The authors indeed provided all raw data on which the study is based. The Excel table with all raw data is provided in a Additional file 1.

## Abbreviations

BFI-10: Big-Five Inventory, 10 items
chi^2^: Value at chi-square distribution of values
Coef: Regression coefficient
df: Degrees of freedom
GSW-6: Generalisierte Selbstwirksamkeit (generalized self-efficacy)
H_0_: No differences hypothesis between model and population
IDAC-4c+: Index of Dental Anxiety and Fear-four dimensions
LS-S: Loneliness Scale-short version
M: Mean
N: Sample size
p: Level of test’s significance, two-tailed value
R^2^: Proportion of variance of dependent variable explained by whole model
SANB-5: Skala Angst vor negative Bewertung (short version of Fear of Negative Evaluation Scale)
SD: Standard deviation
SE: Standard error of estimate
STAI: State-Trait-Anxiety-Inventory, state subscale
Sub A: Skala Subklinische Angst (subclinical anxiety scale)
t: T-value on a t-distribution
Vc: Variation coefficient: (SD/M) × 100
